# Expanding the clinical spectrum of anti-DPPX encephalitis: a multicenter retrospective study

**DOI:** 10.3389/fnins.2024.1379933

**Published:** 2024-05-02

**Authors:** Yining Gao, Yizongheng Zhang, Hangxing Chunyu, Yongfeng Xu, Ying Wang, Suzhi Liu, Jie Chang, Bo Tang, Congying Xu, Yi Lu, Jian Zhou, Xiangyong Kong, Xiaoying Zhu, Sheng Chen, Qinming Zhou, Huanyu Meng

**Affiliations:** ^1^Department of Neurology, Ruijin Hospital, Shanghai Jiao Tong University School of Medicine, Shanghai, China; ^2^Department of Nuclear Medicine, Ruijin Hospital, Shanghai Jiao Tong University School of Medicine, Shanghai, China; ^3^Department of Neurology, The Second Affiliated Hospital, Zhejiang University School of Medicine, Zhejiang, China; ^4^Department of Neurology, Taizhou Hospital of Zhejiang Province, Zhejiang, China; ^5^Department of Neurology, Huadong Hospital Affiliated to Fudan University, Shanghai, China; ^6^Department of Neurology, Affiliated Hangzhou First People’s Hospital School of Medicine, Westlake University, Hangzhou, Zhejiang, China; ^7^Department of Neurology, The Second Hospital of Jiaxing, Jiaxing, Zhejiang, China; ^8^Department of Neurology, The First Affiliated Hospital of Bengbu Medical University, Bengbu, Anhui, China; ^9^Department of Pediatrics, The First People’s Hospital of Yongkang, Yongkang, China; ^10^Department of Neurology, Yongkang Traditional Chinese Medicine Hospital, Zhejiang, China; ^11^Department of Neurology, Shanghai General Hospital, Shanghai Jiao Tong University School of Medicine, Shanghai, China

**Keywords:** anti-DPPX encephalitis, anti-DPPX antibody, multicenter, clinical characteristic, immunotherapy

## Abstract

**Objective:**

Anti-dipeptidyl-peptidase-like protein-6 (DPPX) encephalitis is a rare autoimmune encephalitis, and clinical and experimental information regarding this disease is limited. We conducted this study to comprehensively describe the clinical characteristics, ancillary test results, neuroimaging results, and treatment response in a group of Chinese patients with anti-DPPX encephalitis for better understanding this disease.

**Methods:**

We recruited 14 patients who tested positive for anti-DPPX antibodies in the serum and/or cerebrospinal fluid from 11 medical centers between March 2021 and June 2023. This retrospective study evaluated data on symptoms, autoantibody test, auxiliary examinations, treatments, and outcomes.

**Results:**

The average age at diagnosis was 45.93 ± 4.62 years (range: 11–72 years), and 9 of the 14 patients were males. The main symptoms included cognitive impairment (50.0%, 7/14), central nervous system hyperexcitability (42.9%, 6/14), gastrointestinal dysfunction (35.7%, 5/14), and psychiatric disorders (35.7%, 5/14). Notably, we discovered specific findings on ^18^F-fluorodeoxyglucose positron-emission tomography (PET)/magnetic resonance imaging in two patients. Co-existing autoantibodies were identified in two patients. Parainfection was identified in four patients. One patient had other autoimmune diseases, and one had tumor. Eleven patients received immunotherapy and most patients improved at discharge. Surprisingly, three male patients but no female patients relapsed during the 6 months of follow-up.

**Conclusion:**

The development and outcome of anti-DPPX encephalitis are variable. Male patients were predominant in our cohort. The most common symptoms were the classical triad of prodromal gastrointestinal dysfunction, cognitive and mental disorders, and central nervous system hyperexcitability. Infections, immune dysregulation, and tumors may be important etiologies. Long-term monitoring of disease development should be done in male patients. Overall, our results highlight novel clinical characteristics of anti-DPPX encephalitis.

## 1 Introduction

Anti-dipeptidyl-peptidase-like protein-6 (DPPX) encephalitis is a novel autoimmune disease of the central nervous system (CNS) discovered in 2013, which is characterized by prominent CNS hyperexcitability and unexplained prodromal diarrhea (Boronat et al., 2013). The progression of anti-DPPX encephalitis is often insidious and subacute, and varies among different patients (Tobin et al., 2014). The median age at symptom onset is 52 years, and 63% of affected individuals are male (Xiao et al., 2022). Growing numbers of cases were reported worldwide, expanding the clinical spectrum of anti-DPPX encephalitis. However, there is a lack of reports on this disease in China. Given the limited knowledge of mechanisms of anti-DPPX antibodies and scarce experiences in diagnosis and treatment of anti-DPPX encephalitis, there is an urgent need for more comprehensive studies.

Patients with anti-DPPX encephalitis have antibodies targeting DPPX in the serum or cerebrospinal fluid (CSF) (Boronat et al., 2013). DPPX is a regulatory subunit of the Kv4.2 potassium channels expressed on neuronal cell surface in both the CNS and myenteric plexus (Boronat et al., 2013). Anti-DPPX antibodies can cause decreases in DPPX and Kv4.2, affecting inhibitory currents (Hara et al., 2017). Consequently, patients with anti-DPPX antibodies often present with conditions arising from CNS hyperexcitability such as myoclonus and seizures (Boronat et al., 2013). Other symptoms of anti-DPPX encephalitis include cognitive/mental dysfunction, brainstem or cerebellar disorders, sleep disturbances, and dysautonomia (Hara et al., 2017). Importantly, prodromal gastrointestinal symptoms and weight loss with uncertain reason are also found in most patients (Hara et al., 2017). *In vitro* experiments revealed that patients’ sera and purified IgG increased the excitability and action potential frequency in the enteric nervous system neurons (Piepgras et al., 2015), which could explain the gastrointestinal symptoms. However, the etiology and pathogenesis of anti-DPPX antibodies are still undefined, with a lack of basic studies and clinical analysis.

In this study, we initially conducted a multicenter retrospective analysis of 14 patients with anti-DPPX encephalitis in China. We summarized the clinical indices of Chinese patients and revealed novel findings in symptoms and neuroimaging. Additionally, we used the modified Rankin scale (mRS) scores to evaluate the prognosis in these patients. Our study aims to better understand the characteristics of anti-DPPX encephalitis and provide new insights for diagnosis and treatment.

## 2 Materials and methods

### 2.1 Ethical approval and patient consent

This study was approved by the Ethics Committee of Ruijin Hospital, Shanghai Jiaotong University School of Medicine, ensuring compliance with ethical standards (2021CER375). All procedures were conducted in accordance with the principles outlined in the Declaration of Helsinki. Prior to inclusion, all patients provided informed consent for the use of their medical records, thus ensuring the protection of their privacy and confidentiality throughout the study.

### 2.2 Patients and data collection

Between March 2021 and June 2023, 14 patients with positive anti-DPPX antibodies in the serum or CSF were enrolled. The patients were sourced from 11 medical centers in China (Supplementary material).

**Figure 1:**
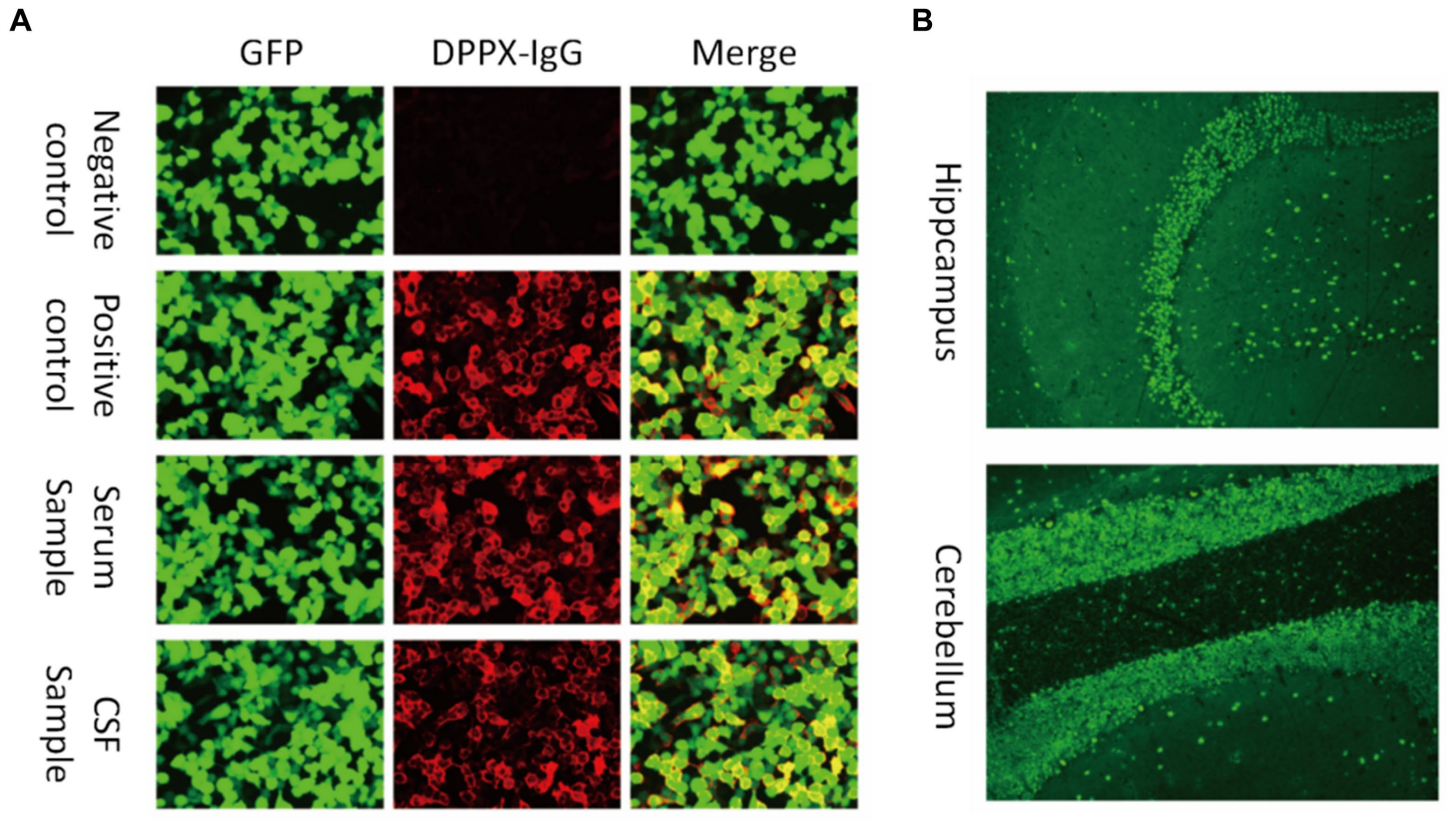
Antibodies against DPPX were detected in both the serum and cerebrospinal fluid (CSF). **(A)** Images show the patient sample in serum and CSF and the negative and positive controls by cell-based assay testing. Green fluorescent protein (GFP) was used to distinguish transfected and non-transfected cells as an internal reference. **(B)** Fluorescence of the stratum molecular of the hippocampus (neuropil staining) and the cerebellum by tissue-based assay.

All autoimmune encephalitis (AE) patients included in this study satisfied the criteria of AE described by Graus et al. (2016) and had a positive result for anti-DPPX antibodies in either the serum or CSF by cell-based assay (CBA) and tissue-based assay (TBA) testing. The diagnoses of all patients were confirmed by two senior attending physicians. The flowchart for patient screening and inclusion is shown in Figure 1.

In this retrospective study, the data on demographics, initial symptoms, primary symptoms, results of auxiliary laboratory tests, cranial magnetic resonance imaging (MRI), ^18^F-fluorodeoxyglucose (FDG) positron-emission tomography (PET)/MRI, immunotherapy regimens, and prognosis were collected at admission, discharge, and follow-up. MRI results were interpreted by two radiologists blinded to the patient’s medical histories. The PET/MRI results were analyzed by two nuclear medicine specialists. Any discrepancies were resolved through a consensus decision involving a third senior radiologist or nuclear medicine physician. Clinical relapse was defined as the new onset or worsening of neurologic symptoms after the initial improvement or stabilization for at least 2 months. The modified Rankin Scale (mRS) was used to evaluate the clinical outcomes. An improvement in the mRS score was defined as an effective response to immunotherapy. Subsequently, the patients were followed up for a duration of 6 months to monitor their progress and treatment outcomes through face-to-face or telephone interviews.

### 2.3 Antibody assay

For antibody detection in all samples, a TBA of monkey brain slices was conducted to examine the pattern of antibody reactivity in the hippocampus and cerebellum. To further examine antibody types and titers, a CBA method was employed, conducted by Dian Diagnostics (Hangzhou, China). HEK293 cells were plated in 96-well plates and co-transfected with full-length human DPPX and pcDNA3.1-EGFP. Then, 36 h later, cells were fixed with 4% paraformaldehyde for 20 min and treated for antibody detection. Cells were incubated with CSF without dilution and serum diluted 1:10 in phosphate-buffered saline (PBS) with 10% goat serum for 2 h at room temperature. The cells were then washed three times with PBS-0.1% Tween 20, incubated for 30 min with goat anti-human IgG (1:500; Thermo Scientific, Waltham, MA), washed again with PBS-0.1% Tween 20, and evaluated using immunofluorescence microscopy. Two independent blinded assessors classified each sample as positive or negative based on the intensity of surface immunofluorescence compared to non-transfected cells and control samples. Once validated, positive specimens were serially diluted from 1:1 to 1:1000 to determine the titers. The final titer was defined as the dilution value at which the specific fluorescence was barely identifiable and expressed as the corresponding dilution value (Ni et al., 2022).

Panels of autoantibodies related to AE, CNS inflammatory demyelinating diseases, and paraneoplastic syndromes were tested. The samples were screened for anti-neural cell (NMDAR, CASPR2, LGI1, AMPAR1, AMPAR2, GABABR, GABAARa1, GABAARg2, GABAARb3, GlyRa1, DPPX, IgLON5, mGluR1, mGluR5, D2R, NRXN3A, KLHL11, GluK2, AK5, VGO, VGCC, AQP4, MOG and GFAP) and onconeural antibodies (Hu, Yo, Ri, Ma2, Ma1, CV2/CRMP5, amphiphysin, Tr/DNER, SOX1, Titin, Zic4, Recoverin, PKC γ and GAD65) using a CBA. We also tested for immunorelated antibodies such as anti-nuclear antibodies.

### 2.4 Statistical analysis

Statistical analyses were performed using GraphPad Prism v8.0 (GraphPad Software, San Diego, CA). Paired *t*-tests were conducted to assess the mRS scores between patient admission, discharge, and follow-up. The unpaired t-test was used to compare mRS scores between sexes at discharge and during follow-up. A significance level of *p* < 0.05 was used to determine if there were significant differences. All statistical tests were two-tailed.

## 3 Results

### 3.1 Clinical manifestations

**Figure 2:**
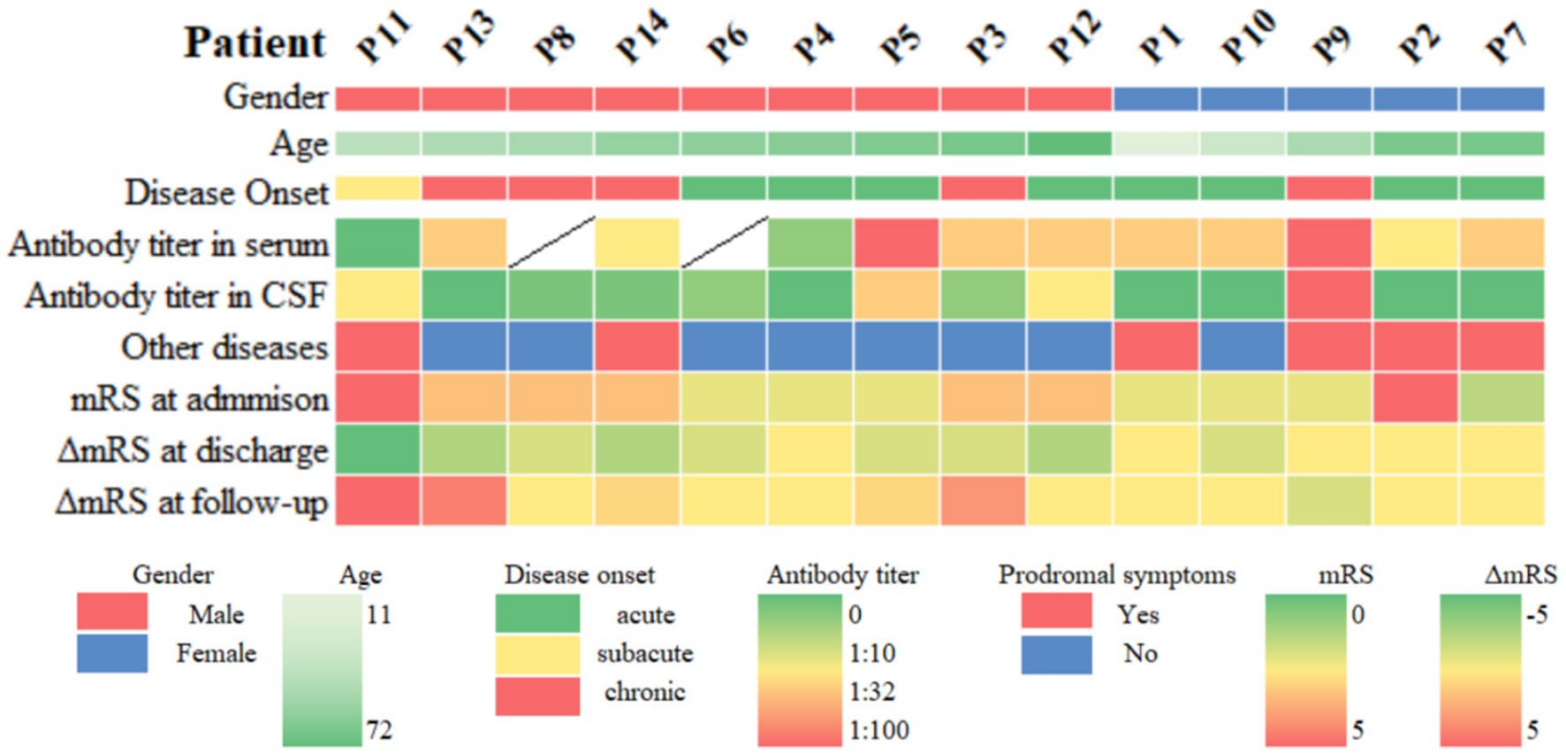
Heatmap of clinical information of patients with anti-DPPX encephalitis. Other diseases include neoplasms, infection, and immune-related factors. ΔmRS at discharge = mRS at discharge − mRS at admission, ΔmRS at follow-up = mRS at follow-up − mRS at discharge. mRS, modified Rankin Scale; CSF, cerebrospinal fluid.

We recruited 14 patients with anti-DPPX encephalitis in serum or CSF, including 9 males and 5 females. The average age at diagnosis was 45.93 ± 4.62 years (range: 11–72 years). There was no significant difference in age between sexes (*p* > 0.05). In general, 9 patients experienced an acute or subacute onset (<3 months), and 5 exhibited a chronic onset (>3 months). The clinical features are summarized in Table 1 and described in further detail in Table 2 and Figure 2.

**Table 1:** Age, gendersex, initial symptoms, primary symptoms, CSF testing, MRI, and mRS scores of patients with anti-DPPX encephalitis.

Characteristics	*N* (%)
Sex Male Female	9/14 64.3% 5/14 35.7%
Age, year Mean (range)	45.93 (11–72)
Initial symptoms Gastrointestinal symptoms Dizziness Ataxia Seizures Cognitive impairment Fever Hallucinations Headache	5/14 35.7% 4/14 28.6% 3/14 21.4% 2/14 14.3% 2/14 14.3% 2/14 14.3% 2/14 14.3% 1/14 7.1%
Primary symptoms Cognitive impairment CNS hyperexcitability Myoclonus Seizures Limb convulsions Gastrointestinal symptoms Psychiatric abnormalities Hallucinations Dizziness Ataxia Weight loss Consciousness changes Nystagmus Sleep disturbances Bulbar dysfunction Weakness in lower limbs	7/14 50.0% 6/14 42.9% 3/6 2/6 1/6 5/14 35.7% 5/14 35.7% 4/14 28.6% 4/14 28.6% 3/14 21.4% 2/14 14.3% 2/14 14.3% 2/14 14.3% 2/14 14.3% 1/14 7.1% 1/14 7.1%
CSF testing Normal Protein >500 mg/L Chloride >130 mmol/L Glucose >4.5 mmol/L Pleocytosis	9/14 64.3% 2/14 14.3% 2/14 14.3% 1/14 7.1% 0/14 0.0%
MRI Normal or nonspecific changes Atrophy or swollen in the hippocampus Cerebellar atrophy Frontotemporal abnormal signals	8/14 57.1% 3/14 21.4% 2/14 14.3% 1/14 7.1%
mRS at admission 2 3 4 5	1/14 7.1% 6/14 42.9% 5/14 35.7% 2/14 14.3%
mRS at discharge 0 1 2 4	1/14 7.1% 7/14 50.0% 5/14 35.7% 1/14 7.1%
mRS at follow-up 0 1 2 5	4/14 28.6% 5/14 35.7% 2/14 14.3% 3/14 21.4%

CSF, cerebrospinal fluid; MRI, magnetic resonance imaging; mRS, modified Rankin Scale; CNS, central nervous system.

**Table 2:** Details of patients with anti-DPPX encephalitis.

Patient number	Patient 1	Patient 2	Patient 3	Patient 4	Patient 5	Patient 6	Patient 7	Patient 8	Patient 9	Patient 10	Patient 11	Patient 12	Patient 13	Patient 14
Sex	Female	Female	Male	Male	Male	Male	Female	Male	Female	Female	Male	Male	Male	Male
Age (years)	11	60	62	54	58	51	62	39	38	22	30	72	36	48
Onset pattern	Acute	Acute	Chronic	Acute	Acute	Acute	Acute	Chronic	Chronic	Acute	Subacute	Acute	Chronic	Chronic
Initial symptoms	Fever, hallucinations, dizziness, headache.	Cognitive impairment	Ataxia, dizziness	Gastrointestinal symptoms	Epilepsy, cognitive impairment	Gastrointestinal symptoms	Fever	Auditory hallucinations	Ataxia, dizziness	Epilepsy	Gastrointestinal symptoms	Gastrointestinal symptoms	Gastrointestinal symptoms	Dizziness, ataxia
Primary symptoms	Psychiatric and behavioral abnormalities	Cognitive impairment, limb convulsions	Cerebellar ataxia	Ataxia, nystagmus	Epilepsy, cognitive impairment	Psychiatric and behavioral abnormalities, nausea, vomiting	Cognitive impairment	Psychiatric and behavioral abnormalities, cognitive impairment, auditory and visual hallucinations	Sleep disturbance, dizziness, weight loss, myodystony	consciousness disorder, epilepsy	Psychiatric and behavioral abnormalities, delirium, myoclonus, hallucinations, cognitive impairment, weight loss, diarrhea	Cognitive impairment, psychiatric and behavioral abnormalities, sleep disorders, hallucinations, abdominal pain, diarrhea	Memory decline, bulbar syndrome, myoclonus, diarrhea	Ataxia, nystagmus, weakness in the lower limbs, dizziness
Neoplasms, infections, and immune-related factors	Upper respiratory tract infection	Breast cancer	(−)	(−)	(−)	(−)	Upper respiratory tract infection	(−)	HSV-1	(−)	Ankylosing spondylitis Rheumatoid arthritis Inflammatory bowel disease	Upper respiratory tract infection	(−)	(−)
CSF testing	Normal	Protein: 91.8 mg/dL	Protein: 57 mg/dL	Normal	Chloride: 133 mmol/L, OCB(+), IgG index:0.94	Normal	Normal	Normal	Normal	Normal	Normal	Glucose: 5.00 mmol/L,OCB(+), IgG index: 0.86	Normal	Chloride: 139 mmol/L
DPPX antibodies in serum	1:100	1:32	1:100	1:10	1:320	NA	1:100	NA	1:320	1:100	(−)	1:100	1:100	1:32
DPPX antibodies in CSF	(−)	(−)	1:10	(−)	1:100	1:10	(−)	1:1	1:32	(−)	1:32	1:32	(−)	1:1
Other positive autoimmune antibodies	(−)	(−)	(−)	(−)	(−)	(−)	(−)	(−)	(−)	(−)	Anti-CASPR2 antibody, 1:10 in CSF	(−)	Anti-NMDAR antibody, 1:32 in serum	(−)
MRI	Normal	Abnormal swelling and enhancement signals in the cortical areas of the left temporal lobe and parieto-occipital lobe.	Normal	Normal	Normal	Normal	Bilateral frontal and parietal white matter, bilateral basal ganglia: Multiple small hyperintense lesions	Normal	Cerebellar atrophy	Hippocampal swelling	Normal	Mild atrophy of the left hippocampus	Normal	Cerebellar atrophy

CSF, cerebrospinal fluid; MRI, magnetic resonance imaging.

The initial symptoms observed in patients consisted of gastrointestinal symptoms (35.7%, 5/14), dizziness (28.6%, 4/14), ataxia (21.4%, 3/14), seizures (14.3%, 2/14), cognitive impairment (14.3%, 2/14), fever (14.3%, 2/14), hallucinations (14.3%, 2/14), and headache (7.1%, 1/14).

During the course of disease, the most common symptoms in patients were cognitive impairment (50.0%, 7/14), CNS hyperexcitability (42.9%, 6/14, including myoclonus [3/6], seizures [2/6], and limb convulsions [1/6]), gastrointestinal symptoms (35.7%, 5/14), and psychiatric abnormalities (35.7%, 5/14). Hallucination was seen in four patients, including auditory and visual hallucinations. In addition, four patients had dizziness, three patients developed ataxia, and two had consciousness changes. Nystagmus (14.3%, 2/14), sleep disturbances (14.3%, 2/14), bulbar dysfunction (7.1%, 1/14) and weakness in the lower limbs (7.1%, 1/14) also occurred. Two patients had unexplained weight loss. The clinical presentation of the 14 patients showed high heterogeneity.

Concurrent diseases were found in six patients. Four patients had preceding infections, including three with upper respiratory tract infections and fever, and one with herpes simplex virus type 1 (HSV-1). One patient had breast cancer. Interestingly, one patient had severe autoimmune diseases, including ankylosing spondylitis, rheumatoid arthritis, and inflammatory bowel disease. He showed unexplained weight loss. Despite full recovery at discharge (mRS = 0), the patient relapsed during follow-up (mRS = 5).

### 3.2 Ancillary tests

**Figure 3:**
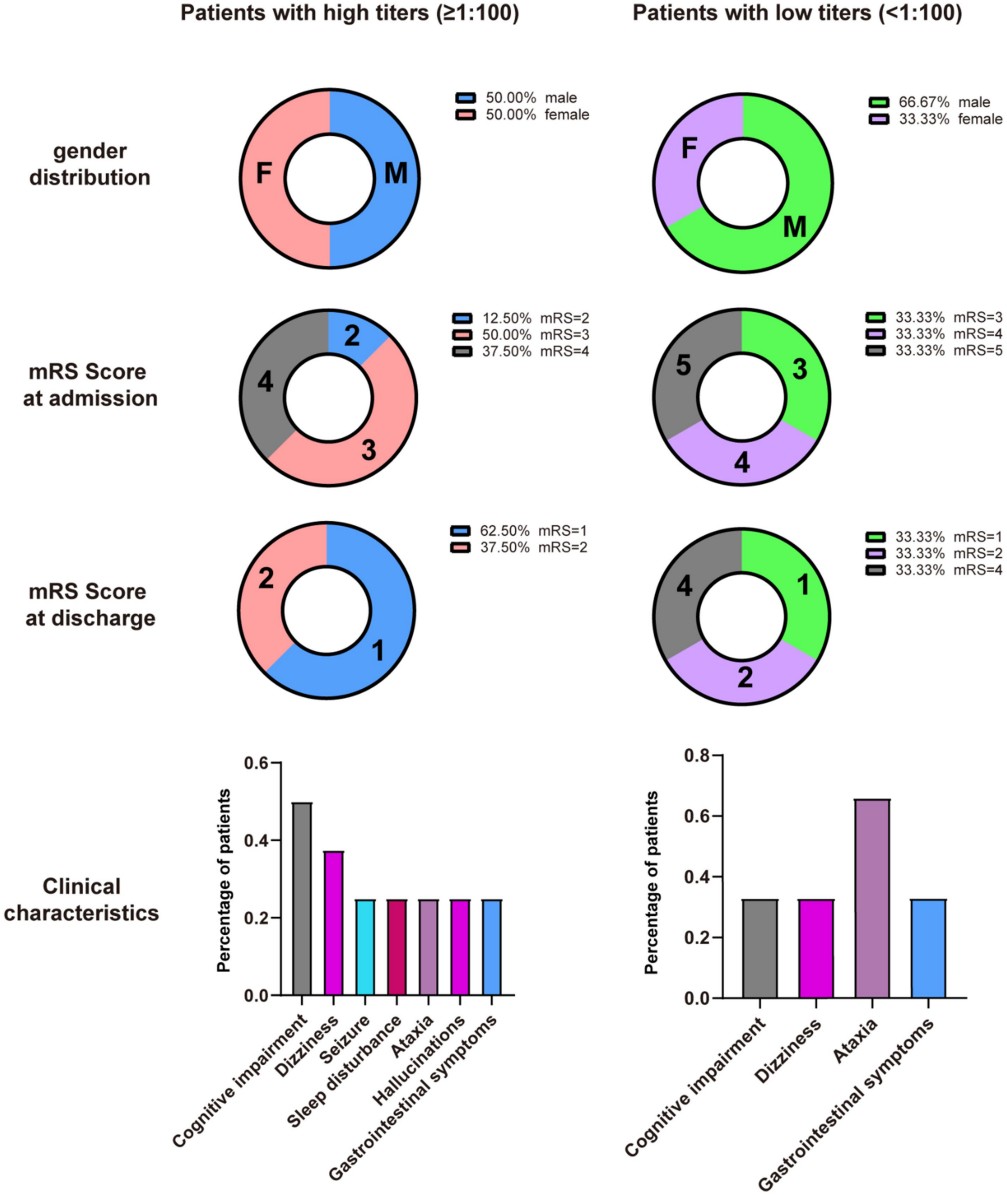
Comparison between patients with high titers (≥1:100) and low titers (<1:100) in serum.

Ancillary test results are summarized in Tables 1, 2. All patients were examined for autoantibodies in the serum or CSF (Figure 1). Serum samples were available in 12 patients, and 11 of them were positive for anti-DPPX antibodies. All patients provided CSF samples, and 7 patients were positive for anti-DPPX antibodies. Five patients tested positive for antibodies to DPPX in both serum and CSF. Interestingly, one patient in our cohort tested negative in serum but positive (1:32) in CSF for anti-DPPX antibodies. According to the serum antibody titers, we divided the patients into a high-titer group (≥1:100) and a low-titer group (<1:100). We then compared sex, clinical manifestations, and mRS scores between these two groups (Figure 3). Two patients had co-existing neural autoantibodies: one with anti-CASPR2 antibody and one with anti-NMDAR antibody.

All patients underwent CSF examination, with five showing abnormal results. Two had elevated protein levels, two had elevated chloride levels, and one had increased glucose levels. However, we did not see any patients with pleocytosis.

**Figure 4:**
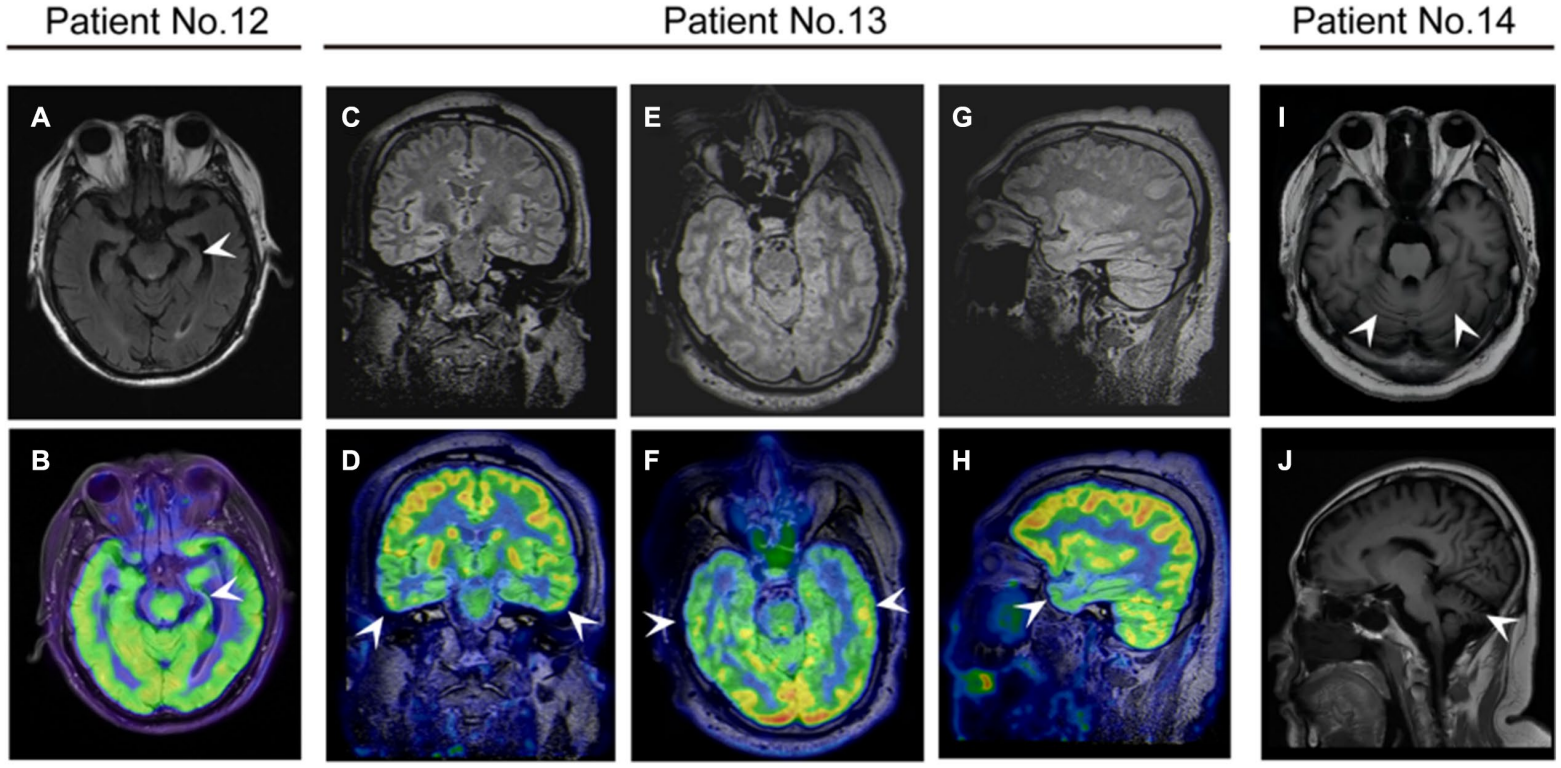
The neuroimaging of patients with anti-DPPX encephalitis. **(A,B)** Patient 12 with cognitive impairment exhibited atrophy of the left hippocampus on magnetic resonance imaging (MRI) and decreased ^18^F-fluorodeoxyglucose (FDG) uptake in the left hippocampus on positron emission tomography (PET)/MRI. Patient 13 with cognitive impairment exhibited decreased ^18^F-FDG uptake in the bilateral temporal lobes on PET/MRI in **(C,D)** coronal **(E,F)** axial, and **(G,H)** sagittal views. **(I,J)** Patient 14 with ataxia showed cerebellar atrophy on MRI.

All patients were examined using cranial MRI, and six (42.9%) showed abnormalities. One patient displayed cortical swelling and enhancement in the left temporal and parietal lobes, and one had multiple high-signal lesions in the bilateral frontal and parietal white matter and basal ganglia. Abnormal signals in the hippocampus were identified in two patients: one with hippocampal swelling and one with hippocampal atrophy. Two patients exhibited cerebellar atrophy (Figure 4). Additionally, two patients underwent ^18^F-FDG PET/MRI, which showed that both had hypometabolism in the temporal lobe (Figure 4). In addition, one of them showed hypometabolism in the thalamus.

### 3.3 Treatment and follow-up

**Figure 5:**
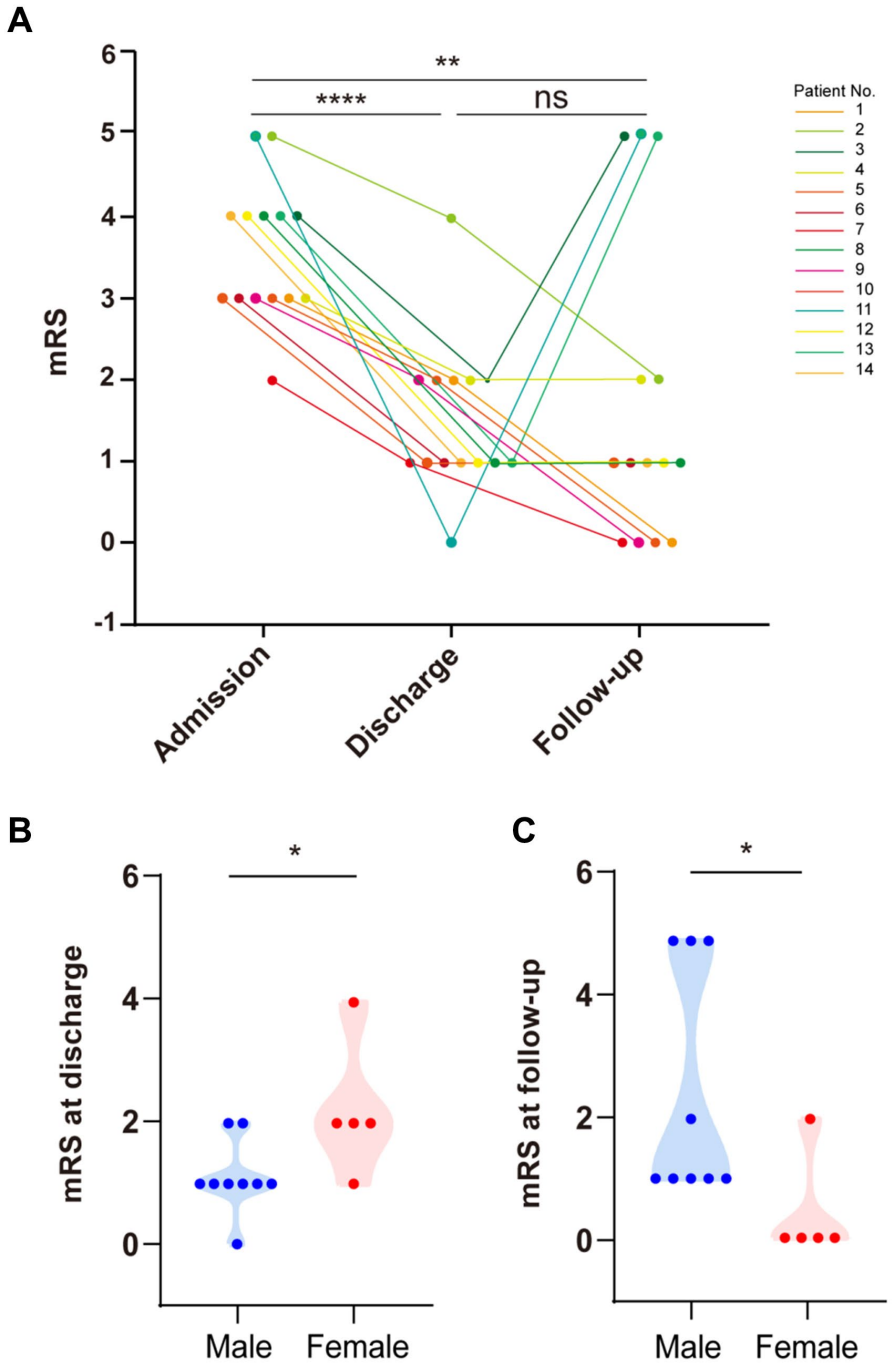
Modified Rankin Scale (mRS) scores at admission, discharge, and follow-up. **(A)** There was a significant difference in mRS scores between patients at admission and discharge (paired *t*-test, *p* < 0.0001). There was a significant difference in mRS scores between patients at admission and follow-up (paired *t*-test, *p* < 0.01). There was no significant difference in mRS scores between patients at discharge and follow-up. **(B)** Females had higher mRS scores than males at discharge (*p* < 0.05). **(C)** Males had higher mRS scores than females during follow-up (*p* < 0.05).

Eleven patients received immunotherapy (including glucocorticoids, intravenous immunoglobulin (IVIg), and azathioprine), six had substantial recovery (mRS = 0–1), four had mild disability (mRS = 2), and one slightly improved (mRS = 4). Notably, three patients without immunotherapy also showed improvement (Table 1). We recorded the mRS scores of all 14 patients during the course of the disease. The average mRS score at admission was 3.57 ± 0.23. There was a significant improvement in the mRS score at discharge compared to that at admission (*p* < 0.05), with an average score of 1.50 ± 0.94 at discharge (Figure 5A).

After discharge, we followed up with all patients for about 6 months. During this period, four patients finally achieved complete recovery with no symptoms of anti-DPPX encephalitis (mRS = 0), seven continuously improved (mRS = 1–3), and three had recurrence of disease (mRS = 5). The average mRS score at follow-up was 1.71 ± 0.50. Overall, there was a significant improvement in the mRS score at follow-up compared to that at admission (*p* < 0.05), but there was no significant difference between the mRS score at follow-up and discharge (*p* = 0.71) (Figure 5A). These results indicated the good effect of immunotherapy and the long-term stableness in most patients of the cohort.

Importantly, the three patients who relapsed during follow-up were all males. We further analyzed the differences in the effectiveness of immunotherapy between sexes (Figure 5). Upon admission, the mRS score showed no significant differences between sexes (*p* = 0.24). However, males presented more favorable outcomes at discharge than females (*p* < 0.05) (Figure 5B). Surprisingly, this tendency was reversed at the 6 months follow-up, with males getting worse scores compared to females (*p* < 0.05) (Figure 5C).

## 4 Discussion

In this study, we conducted the first multicenter study of 14 patients with anti-DPPX encephalitis in China. Most patients were male (64.3%, 9/14), which was similar to previous studies (Xiao et al., 2022). The average age at the time of diagnosis was 45.93 ± 4.62 years, with a broad range from 11 to 72 years, including the youngest case to date.

### 4.1 Clinical characteristics of anti-DPPX encephalitis in our cohort

DPPX is widespread in the hippocampus, cerebellum, striatum, brainstem, and myenteric plexus (Clark et al., 2008), leading to a multifocal neurological phenotype in anti-DPPX encephalitis. Broader symptoms were identified in the following decade. The classical triad of anti-DPPX encephalitis includes prodromal weight loss/gastrointestinal dysfunction, cognitive/mental disorders, and CNS hyperexcitability (Hara et al., 2017). Most patients also exhibited brainstem or cerebellar disorders, sleep disorders, and dysautonomia (Boronat et al., 2013; Tobin et al., 2014). Atypical symptoms, such as seizures, hallucination, allodynia, pruritus, opsoclonus, paresthesia, orobuccolingual dyskinesia, and cardiac dysrhythmia have also been found (Balint et al., 2014; Tobin et al., 2014; Doherty et al., 2017; Wijntjes et al., 2018; Micieli et al., 2019; Deuel et al., 2020; Mbonde et al., 2021; Bjerknes et al., 2022).

We observed both typical and atypical presentations of patients with anti-DPPX encephalitis. Most of our patients exhibited cognitive impairment, CNS hyperexcitability, gastrointestinal dysfunction, and psychiatric disorders, which is consistent with the literature. Ataxia and seizures were not rare in our study. However, weight loss was only seen in two patients. Particularly, visual and auditory hallucinations were found in four patients. We also identified two patients with sleep disorder and one with bulbar syndrome. These symptoms are not commonly seen in anti-DPPX encephalitis. Interestingly, two patients presented epilepsy as initial symptom, which is a rare phenomenon (Bien et al., 2020; Dziadkowiak et al., 2021). Preceding infection was found in four patients, which may be a possible cause of this disease. These results revealed high heterogeneity of anti-DPPX encephalitis, and suggested damages beyond hyperexcitability of anti-DPPX antibodies.

### 4.2 Expanding the imaging spectrum of anti-DPPX encephalitis

Most patients with anti-DPPX encephalitis had normal MRI findings (Hara et al., 2017). Abnormalities in MRI often show non-specific results, such as white matter changes (Boronat et al., 2013; Hara et al., 2017) and microangiopathic periventricular hyperintensity (Hara et al., 2017). ^18^F-FDG PET/MRI was used in several patients, which showed reduced uptake in the caudate nuclei and frontal cortex (Piepgras et al., 2015), increased gastrointestinal tract activity (Stoeck et al., 2015), and asymmetric metabolism in extraocular muscles (Doherty et al., 2017). One study revealed a higher proportion of specific findings on MRI in Chinese patients than in non-Chinese patients (Xiao et al., 2022).

In our study, we found MRI abnormalities in approximately 42.9% of patients, including cortical enhancement, white matter changes, hippocampal swelling or atrophy, and cerebellar atrophy. ^18^F-FDG PET/MRI in two patients demonstrated hypometabolism of the temporal lobe and thalamus. Our results revealed several specific findings consistent with clinical symptoms in patients. Patient 12 presented cognitive impairment and sleep disorders. MRI of the patient showed mild atrophy of the left hippocampus, and ^18^F-FDG PET/MRI showed hypometabolism in the bilateral temporal lobe and thalamus, as previously described (Zhou et al., 2020). Patient 14 had predominant cerebellar ataxia, and his MRI showed typical cerebellar atrophy. The patient started to experience walking instability 18 months prior to enrollment and did not receive any treatment before admission. This finding aroused an awareness of immediate consultation for such symptoms because long-term attacks of anti-DPPX antibodies may result in grave consequence such as neurodegeneration. Previous literature also reported temporal lobe and hippocampal atrophy in a few patients (Hara et al., 2017; Wijntjes et al., 2018; Bien et al., 2020). In addition, cerebral or cerebellar atrophy was detected at last follow-up in some patients (Dominguez et al., 2023). Future studies are needed to clarify the relationship between anti-DPPX antibodies and neurodegeneration.

### 4.3 Infection, immune dysregulation, and neoplasms as predisposing factors for anti-DPPX encephalitis

There are no studies on parainfection causality in anti-DPPX encephalitis. Fever was seen in several patients (Xiao et al., 2022). *Blastocystis hominis* infection was found in the stool of one patient (Wijntjes et al., 2018). In our study, three patients had upper respiratory tract infections with significant fever, and one had HSV-1. We also observed a patient with three other autoimmune diseases, including rheumatoid arthritis, ankylosing spondylitis, and inflammatory bowel disease. Additionally, lupus and type 1 or 2 diabetes mellitus were described in the literature (Dominguez et al., 2023; Wan et al., 2023). Peripheral infection and immune dysregulation may be important etiologies of anti-DPPX encephalitis.

B-cell lymphoma was identified in patients with anti-DPPX antibodies (Tobin et al., 2014; Bressers et al., 2016; Hara et al., 2017; Wijntjes et al., 2018; Xiao et al., 2022), as well as breast adenocarcinoma (Bien et al., 2020), and thyroid carcinoma (Xiao et al., 2022). The incidence of neoplasm is about 15.1% (Xiao et al., 2022). In our study, we also identified one female patient with breast cancer. Importantly, the patient (Patient 2) with breast cancer had brain metastasis and made relatively slow progress after immunotherapy (mRS = 5 on admission and mRS = 4 at discharge). One study described a significant decrease of anti-DPPX antibody titer after tumor resection, while another partly improved and then experienced recurrence (Bien et al., 2020). The presence of tumors may complicate disease progression. It is noted that tumors may be detected long after the appearance of neurological symptoms (Wijntjes et al., 2018; Bien et al., 2020). Neoplasm screening is still necessary in patients during follow up.

### 4.4 Co-existing anti-DPPX antibodies and other autoimmune antibodies

Anti-DPPX antibodies were found to co-exist with anti-AQP4 (Bien et al., 2020), anti-CAPSR2 (Miao et al., 2021), anti-GFAP (Xiao et al., 2022), and anti-CRMP5 antibodies (Wan et al., 2023). Others include thyroid-related antibodies, lupus anticoagulant (LA), antinuclear antibody, and anti-cardiolipin IgM (Miao et al., 2021; Tsai et al., 2021).

In our cohort, two patients had additional co-existing antibodies, including anti-CASPR2 and anti-NMDAR antibodies. Patient 11 tested positive for both anti-DPPX and anti-CASPR2 antibodies in the CSF, with titers of 1:32 and 1:10, respectively. After oral glucocorticoids treatment, the patient’s delirium symptoms are alleviated, but other symptoms remained, the anti-DPPX antibody titer remained at 1:32, and the anti-CASPR2 antibody disappeared. Additionally, considering the patient’s presentation with symptoms of neuromyotonia and hallucinations, as well as gastrointestinal symptoms, we believe that this patient should be diagnosed with anti-DPPX encephalitis. Patient 13 tested positive for both serum anti-DPPX and anti-NMDAR antibodies with titers of 1:100 and 1:32, respectively. The main symptoms in this patient were characteristic of anti-DPPX encephalitis, such as diarrhea, muscle spasms, and abnormal sensations. Additionally, considering that the anti-DPPX antibody titer was higher than that of anti-NMDAR antibodies, it was believed that this patient had anti-DPPX encephalitis. There is currently no large-sample study on the typical clinical manifestations of anti-DPPX encephalitis, further research is still needed.

### 4.5 CSF oligoclonal bands and IgG index in anti-DPPX encephalitis

CSF-specific oligoclonal bands (OCBs) or an elevated CSF IgG index are incorporated into the criteria for “autoantibody-negative but probable autoimmune encephalitis.” OCBs are a valuable clinical indicator for multiple sclerosis and multiple neurological autoimmune diseases. CSF OCBs can also be positive in AE, particularly in higher proportions in anti-NMDAR encephalitis (Xue et al., 2023). A novel study discovered that patients with AE who tested positive for OCBs exhibited higher disease severity (Xue et al., 2023). Additionally, OCB-positive patients often have an elevated IgG index. This suggests that OCB positivity or an elevated CSF IgG index can serve as a risk factor for AE disease severity. A retrospective study has shown that in anti-DPPX encephalitis, the proportion of OCB-positive cases is approximately 25% (Blinder and Lewerenz, 2019), similar to the positivity rate in our patients. There is currently no research on the IgG index of patients with anti-DPPX encephalitis.

### 4.6 Enhanced understanding of immunotherapy response in anti-DPPX encephalitis

Immunotherapy has shown effectiveness in about 68% of patients with anti-DPPX encephalitis (Xiao et al., 2022). Nevertheless, 21.3% patients could relapse, especially during steroid tapering (Xiao et al., 2022; Dominguez et al., 2023).

In our study, we reached the same conclusions. All patients in our cohort improved during hospitalization, regardless of immunotherapy. Males seemed to improve at discharge. However, over the 6 months follow-up period, three males (Patients 3, 11, 13), but no females, encountered clinical relapses (21.4%, 3/14). Patients 3 and 11 had a chronic onset, with predominant cerebellar ataxia and classical triad, respectively. Patients 11 and 13 had co-existing antibodies. In addition, Patient 11 had severe systematic autoimmune disease. Two patients were administered steroids with IVIg or azathioprine, while one (Patient 13) did not receive immunotherapy. All of them improved at first and then deteriorated at the follow-up. The specific reasons for the relapse are unknown. Future studies should focus on examining risk factors and design better strategies to predict recurrence.

Notably, the mRS scores were imbalanced between sexes, with males having lower scores at discharge but higher scores at follow-up. A few factors could be considered through an overview of the clinical information of patients, such as disease course, co-existing antibodies, and concurrent illnesses. We first analyzed the differences in outcomes between males and females using the mRS score. Future studies with a larger sample size should verify this observation.

### 4.7 Pediatric patients with anti-DPPX encephalitis

To date, four children aged from 11 to 17 years have been found with anti-DPPX antibodies in the serum (Miao et al., 2021; Xiao et al., 2022). Compared to other pediatric patients reported in previous literature, the patient in our cohort was the youngest and the only girl. All children had an acute onset. Our patient mainly had prodromal infection and psychiatric disturbances during the disease course, which is also seen in previous cases. It seems that all children presented common symptoms of anti-DPPX encephalitis. Moreover, dizziness was found in our patient, and confusion was found in two former cases (Miao et al., 2021; Xiao et al., 2022). Surprisingly, most children had normal MRI and CSF testing results. Compared to most adult patients, the titers of anti-DPPX antibodies in pediatric patients were higher in the serum, but not in the CSF. Previous cases also found several co-existing antibodies in children including anti-GFAP, anti-CASPR2, anti-TPO, and anti-TG, but not in ours. Immunotherapies showed effectiveness in children with anti-DPPX encephalitis, while our patient did not receive any but partially improved. Future studies should continue to focus on younger patients (Supplemental Table S1).

### 4.8 Limitations

Anti-DPPX encephalitis is rare; therefore, the sample size in our multicenter study is relatively small. Although we have found differences in the clinical features between patients of different genders, such as better immune treatment outcomes in female patients and a higher tendency to relapse in male patients. The conclusion that the prognosis differs between sexes may require a larger sample size for further confirmation. Not all patients underwent enhanced MRI. Currently, there is a lack of evidence supporting the significance of enhanced MRI in the diagnosis and evaluation of anti-DPPX encephalitis. Future studies can improve enhanced MRI testing to clarify its value in diagnosing anti-DPPX encephalitis. This case series is a retrospective study, and the information we collected is limited regarding disease progression and treatments after discharge. Prospective analysis with more patients enrolled is necessary for multifactor risk assessment of clinical outcomes.

## 5 Conclusion

In conclusion, we summarized the clinical indices of 14 Chinese patients with anti-DPPX encephalitis. We expanded the symptom spectrum of anti-DPPX encephalitis. Infection, immune dysregulation, and neoplasms may be possible etiologies of this disease. Most patients can benefit from immunotherapy, while males require long-term strategies for treatment because of higher recurrence rates. Future prospective studies with more detailed designs and extended follow-up periods are required to confirm these findings.

## Data availability statement

The original contributions presented in the study are included in the article/Supplementary material, further inquiries can be directed to the corresponding authors.

## Ethics statement

The studies involving human participants were reviewed and approved by the ethics committee of Ruijin hospital, Shanghai Jiaotong University School of Medicine. Written informed consent to participate in this study was provided by the participants’ legal guardian/next of kin. Written informed consent was obtained from the individuals’ and minors’ legal guardian/next of kin for the publication of any potentially identifiable images or data included in this article.

## Author contributions

YG: Data curation, Formal analysis, Writing – original draft. YZ: Data curation, Formal analysis, Writing – original draft, Methodology, Software. HC: Data curation, Formal analysis, Methodology, Software, Writing – original draft. YX: Data curation, Formal analysis, Writing – original draft. YW: Data curation, Formal analysis, Writing – original draft. SL: Data curation, Formal analysis, Writing – original draft. JC: Data curation, Formal analysis, Writing – original draft. BT: Data curation, Formal analysis, Writing – original draft. CX: Data curation, Formal analysis, Writing – original draft. YL: Data curation, Formal analysis, Writing – original draft. JZ: Data curation, Formal analysis, Writing – original draft. XK: Data curation, Formal analysis, Writing – original draft. XZ: Data curation, Formal analysis, Writing – original draft. QZ: Conceptualization, Writing – review & editing. HM: Conceptualization, Writing – review & editing. SC: Data curation, Formal analysis, Writing – review & editing.
