# Supplementary material for: Expanding the clinical spectrum of anti-DPPX encephalitis: a multicenter retrospective study
Source: Front Neurosci. 2024 May 2;18:1379933. doi: 10.3389/fnins.2024.1379933 (PMC11098017; doi:10.3389/fnins.2024.1379933)
Supplement: Supplementary file 1 [file Data_Sheet_1.docx]

Supplementary Material

**Supplementary Table 1.** The 11 medical centers.

The patients were sourced from 11 medical centers in China: Ruijin Hospital, The First People’s Hospital of Shanghai, The First People’s Hospital of Yongkang City in Zhejiang Province, The First People’s Hospital of Hangzhou City, Taizhou Hospital, Yongkang Traditional Chinese Medicine Hospital, The First Affiliated Hospital of Bengbu Medical College, Taizhou Hospital in Zhejiang Province, Huadong Hospital and The Second Affiliated Hospital of Zhejiang University.

**Supplementary Table 2.** The clinical characteristics of pediatric patients with anti-DPPX antibodies.

|  | Xiao et al^1^ ***P5*** | Miao et al^2^ ***P2*** | Miao et al^2^ ***P4*** | This study ***P1*** |
| --- | --- | --- | --- | --- |
| Sex | Male | Male | Male | Female |
| Age | 14 | 14 | 17 | 11 |
| Onset pattern | Acute | Acute | Acute | Acute |
| Primary symptoms | Fever, gastroparesis and constipation | Psychiatric disturbances | Headache | Fever, hallucinations, dizziness, headache |
| Main symptoms | Confusion, cognitive disorder, limb paralysis, respiratory failure, hyperreflexia | Amnesia, confusion | Migrating myoclonus | Psychiatric and behavioral abnormalities |
| MRI | Extensive abnormal signals in the brainstem, basal ganglia and bilateral white matter | Normal | Normal | Normal |
| CSF | (March 2019)  WBC: 220×10^6^/L, Protein: 209.6 mg/dL  (May 2019)  WBC: 10×10^6^/L, Protein: 46.9 mg/dL | Protein: 63 mg/dL | Normal | Normal |
| DPPX antibody | Serum: 1:320  CSF (-) | Serum: 1:100  CSF (-) | Serum: 1:100  CSF (-) | Serum: 1:100  CSF (-) |
| Other antibody | Anti GFAP:  Serum: 1:320,  CSF: 1:10 | Anti TPO: >600IU/ml;  Anti-TG: 345.03IU/ml | Anti-CASPR2: serum: 1:10 | (-) |
| Immunotherapy | IVMP, IVIG | IVIG, methylprednisolone | IVIG, methylprednisolone | None |
| Outcome | partially improved | partially improved | partially improved | partially improved |

DPPX dipeptidyl-peptidase-like protein-6; GFAP: glial fibrillary acidic protein; TPO: thyroidperoxidase; TG: thyroglobulin; CASPR2: contactin-associatedprotein-like2; IVMP: intravenous methylprednisolone; IVIG: intravenous immunoglobulins
